# *trans*-Bis(acridine-κ*N*)dichloridopalladium(II)

**DOI:** 10.1107/S1600536811053335

**Published:** 2011-12-17

**Authors:** Kwang Ha

**Affiliations:** a School of Applied Chemical Engineering, The Research Institute of Catalysis, Chonnam National University, Gwangju 500-757, Republic of Korea

## Abstract

In the title complex, [PdCl_2_(C_13_H_9_N)_2_], the Pd^II^ ion is four-coordinated in an essentially square-planar environment by two N atoms from two acridine ligands and two Cl^−^ anions. The Pd atom is located on an inversion centre, and thus the asymmetric unit contains one half of the complex and the PdN_2_Cl_2_ unit is exactly planar. The dihedral angle between the PdN_2_Cl_2_ unit and the acridine ligand is 84.66 (6)°. In the crystal, the complex mol­ecules are stacked in columns along the *a* axis connected by C—H⋯Cl hydrogen bonds, forming chains along [110]. In the columns, numerous inter­molecular π–π inter­actions between the six-membered rings are present, the shortest ring centroid–centroid distance being 3.722 (4) Å.

## Related literature

For the related crystal structures [Pd*X*_2_(acr)_2_] (*X* = Br, I), see: Ha (2010*a* ▶,*b* ▶).
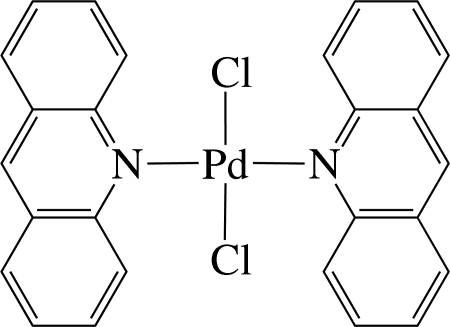


## Experimental

### 

#### Crystal data

[PdCl_2_(C_13_H_9_N)_2_]
*M* *_r_* = 535.72
Triclinic, 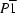

*a* = 8.2114 (16) Å
*b* = 8.8910 (18) Å
*c* = 9.0105 (18) Å
α = 66.188 (4)°
β = 77.230 (4)°
γ = 66.885 (4)°
*V* = 551.99 (19) Å^3^
*Z* = 1
Mo *K*α radiation
μ = 1.10 mm^−1^
*T* = 200 K
0.20 × 0.12 × 0.09 mm

#### Data collection

Bruker SMART 1000 CCD diffractometer
Absorption correction: multi-scan (*SADABS*; Bruker, 2000 ▶) *T*_min_ = 0.679, *T*_max_ = 1.000
3488 measured reflections
2124 independent reflections
1626 reflections with *I* > 2σ(*I*)
*R*_int_ = 0.056

#### Refinement

*R*[*F* ^2^ > 2σ(*F* ^2^)] = 0.059
*wR*(*F* ^2^) = 0.107
*S* = 0.98
2124 reflections
142 parameters
H-atom parameters constrained
Δρ_max_ = 0.83 e Å^−3^
Δρ_min_ = −0.66 e Å^−3^

### 

Data collection: *SMART* (Bruker, 2000 ▶); cell refinement: *SAINT* (Bruker, 2000 ▶); data reduction: *SAINT*; program(s) used to solve structure: *SHELXS97* (Sheldrick, 2008 ▶); program(s) used to refine structure: *SHELXL97* (Sheldrick, 2008 ▶); molecular graphics: *ORTEP-3* (Farrugia, 1997 ▶) and *PLATON* (Spek, 2009 ▶); software used to prepare material for publication: *SHELXL97*.

## Supplementary Material

Crystal structure: contains datablock(s) global, I. DOI: 10.1107/S1600536811053335/bq2326sup1.cif

Structure factors: contains datablock(s) I. DOI: 10.1107/S1600536811053335/bq2326Isup2.hkl

Additional supplementary materials:  crystallographic information; 3D view; checkCIF report

## Figures and Tables

**Table:** 

Pd1—N1	2.055 (4)
Pd1—Cl1	2.2975 (15)

**Table:** 

N1—Pd1—Cl1	89.75 (12)

**Table 2:** Hydrogen-bond geometry (Å, °)

*D*—H⋯*A*	*D*—H	H⋯*A*	*D*⋯*A*	*D*—H⋯*A*
C5—H5⋯Cl1^i^	0.95	2.74	3.589 (6)	149

Symmetry code: (i) 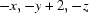.

**Fig. 1.**
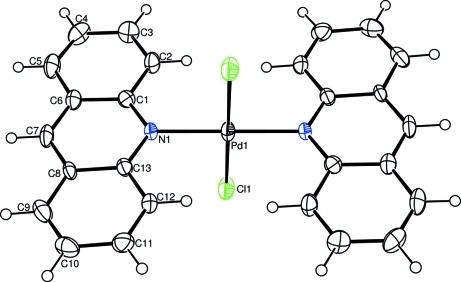
The structure of the title complex, with displacement ellipsoids drawn at the 50% probability level for non-H atoms. Unlabelled atoms are related to the reference atoms by the (1 - x, 1 - y, -z) symmetry transformation.

**Fig. 2.**
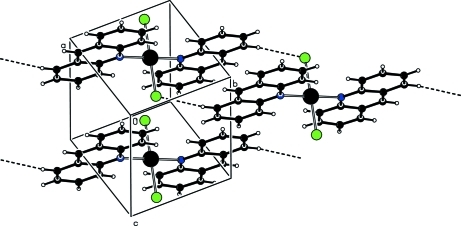
View of the unit-cell contents of the title complex. Hydrogen-bond interactions are drawn with dashed lines.

## supplementary crystallographic information

### Comment

In the title complex, [PdCl_2_(acr)_2_] (acr = acridine, C_13_H_9_N), the Pd^II^ ion is four-coordinated in an essentially square-planar environment by two N atoms from two acridine (acr) ligands and two Cl^-^ anions (Fig. 1 and Table 1). The complex and the iodo analogue [PdI_2_(acr)_2_] crystallized in the triclinic space group *P*1, whereas the analogous bromo Pd(II) complex [PdBr_2_(acr)_2_] crystallized in the monoclinic space group *C*2/*c* (Ha, 2010*a*,*b*).

The Pd atom is located on an inversion centre, and thus the asymmetric unit contains one half of the complex and the PdN_2_Cl_2_ unit is exactly plane. The nearly planar acridine ligands, with a maximum deviation of 0.033 (4) Å from the least-squares plane, are parallel. The dihedral angle between the PdN_2_Cl_2_ unit and acridine ligand is 84.66 (6)°. The Cl atoms are in *trans* conformation with respect to each other and almost perpendicular to the acridine planes, with the bond angle N1—Pd1—Cl1 = 89.75 (12)°. In the crystal, the complex molecules are stacked in columns along the *a* axis and connected by C—H···Cl hydrogen bonds, forming chains along [110]. In the columns, numerous intermolecular π-π interactions between the six-membered rings are present, the shortest ring centroid-centroid distance being 3.722 (4) Å.

### Experimental

To a solution of Na_2_PdCl_4_ (0.2014 g, 0.685 mmol) in H_2_O (20 ml) was added acridine (0.2561 g, 1.429 mmol), and the mixture was refluxed for 7 h. The precipitate was then separated by filtration, washed with acetone and pentane, and dried at 50 °C, to give a yellow powder (0.3369 g). Crystals suitable for X-ray analysis were obtained by slow evaporation from a CH_3_CN solution.

### Refinement

H atoms were positioned geometrically and allowed to ride on their respective parent atoms [C—H = 0.95 Å and *U*_iso_(H) = 1.2*U*_eq_(C)]. The highest peak (0.83 e Å^-3^) and the deepest hole (-0.66 e Å^-3^) in the difference Fourier map are located 1.17 and 0.85 Å from the Pd1 atom, respectively.

### Crystal data

**Table:**

[PdCl_2_(C_13_H_9_N)_2_]	*Z* = 1
*M**_r_* = 535.72	*F*(000) = 268
Triclinic, *P*1	*D*_x_ = 1.612 Mg m^−^^3^
Hall symbol: -P 1	Mo *K*α radiation, λ = 0.71073 Å
*a* = 8.2114 (16) Å	Cell parameters from 940 reflections
*b* = 8.8910 (18) Å	θ = 2.7–22.5°
*c* = 9.0105 (18) Å	µ = 1.10 mm^−^^1^
α = 66.188 (4)°	*T* = 200 K
β = 77.230 (4)°	Block, yellow
γ = 66.885 (4)°	0.20 × 0.12 × 0.09 mm
*V* = 551.99 (19) Å^3^	

### Data collection

**Table:**

Bruker SMART 1000 CCD diffractometer	2124 independent reflections
Radiation source: fine-focus sealed tube	1626 reflections with *I* > 2σ(*I*)
graphite	*R*_int_ = 0.056
φ and ω scans	θ_max_ = 26.0°, θ_min_ = 2.5°
Absorption correction: multi-scan (*SADABS*; Bruker, 2000)	*h* = −10→10
*T*_min_ = 0.679, *T*_max_ = 1.000	*k* = −9→10
3488 measured reflections	*l* = −10→11

### Refinement

**Table:**

Refinement on *F*^2^	Primary atom site location: structure-invariant direct methods
Least-squares matrix: full	Secondary atom site location: difference Fourier map
*R*[*F*^2^ > 2σ(*F*^2^)] = 0.059	Hydrogen site location: inferred from neighbouring sites
*wR*(*F*^2^) = 0.107	H-atom parameters constrained
*S* = 0.98	*w* = 1/[σ^2^(*F*_o_^2^) + (0.0328*P*)^2^] where *P* = (*F*_o_^2^ + 2*F*_c_^2^)/3
2124 reflections	(Δ/σ)_max_ < 0.001
142 parameters	Δρ_max_ = 0.83 e Å^−^^3^
0 restraints	Δρ_min_ = −0.66 e Å^−^^3^

### Special details

**Table:**

Geometry. All e.s.d.'s (except the e.s.d. in the dihedral angle between two l.s. planes) are estimated using the full covariance matrix. The cell e.s.d.'s are taken into account individually in the estimation of e.s.d.'s in distances, angles and torsion angles; correlations between e.s.d.'s in cell parameters are only used when they are defined by crystal symmetry. An approximate (isotropic) treatment of cell e.s.d.'s is used for estimating e.s.d.'s involving l.s. planes.
Refinement. Refinement of *F*^2^ against ALL reflections. The weighted *R*-factor *wR* and goodness of fit *S* are based on *F*^2^, conventional *R*-factors *R* are based on *F*, with *F* set to zero for negative *F*^2^. The threshold expression of *F*^2^ > σ(*F*^2^) is used only for calculating *R*-factors(gt) *etc*. and is not relevant to the choice of reflections for refinement. *R*-factors based on *F*^2^ are statistically about twice as large as those based on *F*, and *R*- factors based on ALL data will be even larger.

### Fractional atomic coordinates and isotropic or equivalent isotropic displacement parameters (Å^2^)

**Table:**

	*x*	*y*	*z*	*U*_iso_*/*U*_eq_	
Pd1	0.5000	0.5000	0.0000	0.0284 (2)	
Cl1	0.27624 (19)	0.42136 (19)	0.1805 (2)	0.0413 (4)	
N1	0.3976 (5)	0.7417 (5)	0.0224 (5)	0.0239 (10)	
C1	0.2822 (7)	0.8767 (7)	−0.0814 (7)	0.0287 (13)	
C2	0.2416 (7)	0.8596 (8)	−0.2178 (7)	0.0343 (15)	
H2	0.2955	0.7513	−0.2350	0.041*	
C3	0.1271 (7)	0.9955 (8)	−0.3232 (8)	0.0399 (16)	
H3	0.1017	0.9806	−0.4132	0.048*	
C4	0.0448 (7)	1.1580 (8)	−0.3032 (8)	0.0438 (17)	
H4	−0.0363	1.2512	−0.3785	0.053*	
C5	0.0810 (7)	1.1817 (8)	−0.1766 (8)	0.0407 (16)	
H5	0.0246	1.2920	−0.1634	0.049*	
C6	0.2011 (7)	1.0455 (7)	−0.0640 (7)	0.0302 (14)	
C7	0.2430 (7)	1.0626 (7)	0.0674 (7)	0.0352 (15)	
H7	0.1892	1.1714	0.0838	0.042*	
C8	0.3604 (7)	0.9262 (7)	0.1749 (7)	0.0254 (13)	
C9	0.4058 (8)	0.9384 (9)	0.3117 (8)	0.0411 (16)	
H9	0.3519	1.0450	0.3321	0.049*	
C10	0.5234 (9)	0.8029 (9)	0.4133 (8)	0.0439 (17)	
H10	0.5517	0.8142	0.5040	0.053*	
C11	0.6047 (8)	0.6432 (8)	0.3845 (7)	0.0407 (16)	
H11	0.6886	0.5478	0.4557	0.049*	
C12	0.5641 (8)	0.6259 (7)	0.2572 (7)	0.0350 (15)	
H12	0.6208	0.5179	0.2397	0.042*	
C13	0.4400 (7)	0.7629 (7)	0.1489 (7)	0.0269 (13)	

### Atomic displacement parameters (Å^2^)

**Table:**

	*U*^11^	*U*^22^	*U*^33^	*U*^12^	*U*^13^	*U*^23^
Pd1	0.0283 (4)	0.0212 (4)	0.0363 (4)	−0.0025 (3)	−0.0064 (3)	−0.0144 (3)
Cl1	0.0379 (9)	0.0295 (9)	0.0540 (11)	−0.0098 (7)	0.0061 (8)	−0.0188 (8)
N1	0.024 (2)	0.020 (2)	0.028 (3)	−0.005 (2)	−0.001 (2)	−0.011 (2)
C1	0.023 (3)	0.030 (3)	0.029 (3)	−0.007 (3)	0.001 (3)	−0.009 (3)
C2	0.030 (3)	0.028 (3)	0.042 (4)	−0.001 (3)	−0.006 (3)	−0.017 (3)
C3	0.033 (3)	0.046 (4)	0.043 (4)	−0.011 (3)	−0.017 (3)	−0.013 (3)
C4	0.025 (3)	0.035 (4)	0.054 (5)	0.000 (3)	−0.012 (3)	−0.003 (3)
C5	0.029 (3)	0.030 (4)	0.059 (5)	−0.003 (3)	0.002 (3)	−0.021 (3)
C6	0.026 (3)	0.024 (3)	0.041 (4)	−0.006 (3)	0.000 (3)	−0.015 (3)
C7	0.031 (3)	0.023 (3)	0.051 (4)	−0.007 (3)	0.010 (3)	−0.020 (3)
C8	0.025 (3)	0.025 (3)	0.029 (3)	−0.011 (3)	0.008 (3)	−0.016 (3)
C9	0.045 (4)	0.047 (4)	0.043 (4)	−0.023 (4)	0.013 (3)	−0.028 (4)
C10	0.061 (4)	0.057 (5)	0.033 (4)	−0.039 (4)	0.000 (4)	−0.018 (3)
C11	0.057 (4)	0.037 (4)	0.032 (4)	−0.022 (3)	−0.010 (3)	−0.007 (3)
C12	0.050 (4)	0.022 (3)	0.035 (4)	−0.010 (3)	−0.008 (3)	−0.012 (3)
C13	0.029 (3)	0.027 (3)	0.029 (3)	−0.009 (3)	0.002 (3)	−0.016 (3)

### Geometric parameters (Å, °)

**Table:**

Pd1—N1^i^	2.055 (4)	C5—H5	0.9500
Pd1—N1	2.055 (4)	C6—C7	1.382 (8)
Pd1—Cl1^i^	2.2975 (15)	C7—C8	1.373 (7)
Pd1—Cl1	2.2975 (15)	C7—H7	0.9500
N1—C1	1.344 (6)	C8—C9	1.420 (7)
N1—C13	1.362 (6)	C8—C13	1.432 (7)
C1—C2	1.420 (7)	C9—C10	1.344 (8)
C1—C6	1.442 (7)	C9—H9	0.9500
C2—C3	1.350 (7)	C10—C11	1.416 (8)
C2—H2	0.9500	C10—H10	0.9500
C3—C4	1.402 (8)	C11—C12	1.344 (7)
C3—H3	0.9500	C11—H11	0.9500
C4—C5	1.353 (8)	C12—C13	1.406 (7)
C4—H4	0.9500	C12—H12	0.9500
C5—C6	1.408 (8)		
			
N1^i^—Pd1—N1	180.0	C7—C6—C5	123.4 (5)
N1^i^—Pd1—Cl1^i^	89.75 (12)	C7—C6—C1	117.3 (5)
N1—Pd1—Cl1^i^	90.25 (12)	C5—C6—C1	119.4 (5)
N1^i^—Pd1—Cl1	90.25 (12)	C8—C7—C6	121.5 (5)
N1—Pd1—Cl1	89.75 (12)	C8—C7—H7	119.2
Cl1^i^—Pd1—Cl1	180.00 (8)	C6—C7—H7	119.2
C1—N1—C13	119.7 (4)	C7—C8—C9	123.2 (5)
C1—N1—Pd1	120.7 (3)	C7—C8—C13	118.6 (5)
C13—N1—Pd1	119.6 (3)	C9—C8—C13	118.2 (5)
N1—C1—C2	120.6 (5)	C10—C9—C8	121.6 (6)
N1—C1—C6	122.0 (5)	C10—C9—H9	119.2
C2—C1—C6	117.4 (5)	C8—C9—H9	119.2
C3—C2—C1	120.7 (5)	C9—C10—C11	119.8 (6)
C3—C2—H2	119.7	C9—C10—H10	120.1
C1—C2—H2	119.7	C11—C10—H10	120.1
C2—C3—C4	121.7 (6)	C12—C11—C10	120.4 (6)
C2—C3—H3	119.1	C12—C11—H11	119.8
C4—C3—H3	119.1	C10—C11—H11	119.8
C5—C4—C3	119.8 (6)	C11—C12—C13	122.0 (5)
C5—C4—H4	120.1	C11—C12—H12	119.0
C3—C4—H4	120.1	C13—C12—H12	119.0
C4—C5—C6	121.0 (5)	N1—C13—C12	121.2 (5)
C4—C5—H5	119.5	N1—C13—C8	120.9 (5)
C6—C5—H5	119.5	C12—C13—C8	118.0 (5)
			
Cl1^i^—Pd1—N1—C1	87.0 (4)	C5—C6—C7—C8	179.7 (5)
Cl1—Pd1—N1—C1	−93.0 (4)	C1—C6—C7—C8	1.6 (8)
Cl1^i^—Pd1—N1—C13	−96.5 (4)	C6—C7—C8—C9	−179.6 (5)
Cl1—Pd1—N1—C13	83.5 (4)	C6—C7—C8—C13	−0.2 (8)
C13—N1—C1—C2	177.5 (5)	C7—C8—C9—C10	−179.0 (6)
Pd1—N1—C1—C2	−6.0 (7)	C13—C8—C9—C10	1.5 (8)
C13—N1—C1—C6	0.2 (8)	C8—C9—C10—C11	0.0 (9)
Pd1—N1—C1—C6	176.8 (4)	C9—C10—C11—C12	−0.6 (9)
N1—C1—C2—C3	−179.3 (5)	C10—C11—C12—C13	−0.4 (9)
C6—C1—C2—C3	−2.0 (8)	C1—N1—C13—C12	−178.2 (5)
C1—C2—C3—C4	0.3 (9)	Pd1—N1—C13—C12	5.2 (7)
C2—C3—C4—C5	0.7 (10)	C1—N1—C13—C8	1.3 (8)
C3—C4—C5—C6	0.2 (9)	Pd1—N1—C13—C8	−175.2 (4)
C4—C5—C6—C7	180.0 (6)	C11—C12—C13—N1	−178.6 (6)
C4—C5—C6—C1	−2.0 (9)	C11—C12—C13—C8	1.9 (9)
N1—C1—C6—C7	−1.7 (8)	C7—C8—C13—N1	−1.4 (8)
C2—C1—C6—C7	−179.0 (5)	C9—C8—C13—N1	178.1 (5)
N1—C1—C6—C5	−179.9 (5)	C7—C8—C13—C12	178.1 (5)
C2—C1—C6—C5	2.8 (8)	C9—C8—C13—C12	−2.4 (8)

Symmetry codes: (i) −*x*+1, −*y*+1, −*z*.

### Hydrogen-bond geometry (Å, °)

**Table:**

*D*—H···*A*	*D*—H	H···*A*	*D*···*A*	*D*—H···*A*
C5—H5···Cl1^ii^	0.95	2.74	3.589 (6)	149.

Symmetry codes: (ii) −*x*, −*y*+2, −*z*.
